# Supplementary material for: Gender sensitivity and stereotypes in medical university students: An Italian cross-sectional study
Source: PLoS One. 2022 Jan 6;17(1):e0262324. doi: 10.1371/journal.pone.0262324 (PMC8735594; doi:10.1371/journal.pone.0262324)
Supplement: Attachment — Submitted filename: DR AJOKE REVIEW.docx [file pone.0262324.s001.docx]

PLOS ONE: Are 2020’s medical students still suffering from gender stereotypes? An Italian crossectional

study

| Pages | Queries | Response |
| --- | --- | --- |
| 3 | In many diseases, such as coronary artery disease, Parkinson disease, irritable bowel  syndrome, neck pain, knee joint arthrosis and tuberculosis, men are treated more extensively  than women (8).  Research found that physicians are more likely to consider symptoms reported by men as organic (8).  Gender awareness in doctors aims to equity and equality in health | Did you mean to say these diseases are treated more extensively by men than women?  Kindly explain and paraphrase your sentence properly.  Sentence structure paraphrasing needed |
| 5 | Method section  A sample of 1258 students received an email with  the background, | The reason/s behind adopting this particular study design  Kindly explain the Sampling techniques for example strategy used for calculation and selection of the sample size.  Explanation about pilot study conducted |
|  |  |  |
|  |  |  |
|  |  |  |
|  |  |  |
|  |  |  |
