# Supplementary material for: Gender sensitivity and stereotypes in medical university students: An Italian cross-sectional study
Source: PLoS One. 2022 Jan 6;17(1):e0262324. doi: 10.1371/journal.pone.0262324 (PMC8735594; doi:10.1371/journal.pone.0262324)
Supplement: Attachment — Submitted filename: Response to Reviewers .docx [file pone.0262324.s003.docx]

Dear Editor,

We are submitting the revised version of our manuscript **“Are 2020’s medical students still suffering from gender stereotypes? An Italian cross-sectional study”**, according to the comments of the reviewers.

**Rev 1:**

We would like to thank you for your useful considerations.

**Rev 2:**

1. **I will suggest that the authors give a proper description of the variables that were used for measurement in the analysis.**

Thank you for this useful suggestion. We added the following paragraph in the Materials and Methods section:

“This paper focuses on the fourth part of questionnaire, assessing gender sensitivity and gender stereotypes in medical students and their association with socio-demographic features, gender medicine knowledge and training experience regarding gender medicine.

Table 1 displays the variables assessed to describe the sample, stratified by gender. In particular, we explored the following characteristics: age, year of course, nationality, marital status, having children, self-perceived health status, having one or more chronic diseases, familiarity for chronic diseases. Self-perceived health status was assessed with a Likert scale, ranging from 1 (very bad) to 5 (very good), and it was subsequently dichotomized in “not good” (score 1 and 2) and “good/very good” (score 3, 4 and 5). Gender medicine knowledge was assessed with questions regarding the correct definitions of sex, gender and gender medicine, gender-related epidemiology of frequent diseases, and true/false questions regarding specific gender medicine issues.

Personal experience during academic training were assessed asking the students whether they have dealt with gender medicine issues during lessons and during traineeships in the wards”.

2. **Tables 1, 3, 4 and 5 in the results section have the same questions. This makes it difficult to ascertain the variables that are being measured and how the results were obtained. If possible, the authors should properly distinguish between the tables to make for easier understanding of the results.**

Thank you for this suggestion. However, we think that a modification of the tables is not necessary, as they report the results of different analysis. In particular, table 1 reports the description of the sample, while tables 3, 4 and 5 report the results of the multivariable regression model exploring the potential predictors of Gender Sensitivity (table 3), GRIP (table 4) and GRID (table 5), as specified in the text.

3. **I will also suggest that the authors provide a more detailed exploration of gender sensitivity and gender role ideology using, if possible, a theoretical framework.**

Thank you for your comment. We modified as follow:

“Gender sensitivity includes the awareness that gender has an impact on health, and affects the presentation of health complaints. This sensitivity allows healthcare professionals to effectively address gender and improve care for both men and women (13). Gender-role ideology represents a health care worker’s attitude towards male and female patients and doctors (14). Furthermore gender-role ideology is present at different levels in health services. Gender role ideology towards patients are a risk factor for inadequate care. For example, several studies show that doctors often attribute psychological symptoms to women for the same symptoms reported (15). Gender role ideology towards doctors instead refer to false beliefs such as thinking that female doctors are more empathic than male doctors (16).”

4. **The authors should include a flow chart to show how participants were recruited into the study.**

Thank you for this comment. As described in the Materials and Methods section we sent the e-mail with the link to online questionnaire to all the medical students of the fourth, fifth year and outside prescribed times, with no exclusion criteria. A total of 1258 students received the e-mail and 430 of them filled in the questionnaire, thus representing our study population. We think that a flow chart would not add any other relevant information on the recruiting process.

5. **The statement "With 63 out of 100 points, Italy ranks 14 in the EU on the Gender Equality Index...." in the Discussion section should be appropriately referenced.**

Thank you, we inserted the correct reference.

6. **The word "Globally" in the results section should be replaced with another word that describes the context of the study population.**

We replaced the word “Globally” with this sentence: “A sample of 430 students completed the questionnaire correctly.”

**Rev 3**

1. **It is suggested that the ethical approval reference number assigned to the study protocol on which data this paper is based be provided in the last sentence of the first paragraph of the materials and methods section of the manuscript.**

Thank you for your comment. We cannot provide a study protocol number because we used the Internal Review Board of the Department of Public Health Sciences, University of Turin.

2. **It would be good if the authors could add the age range of the participants and their socio-demographic characteristics in Table 1.**

Thank you for this remark. We inserted the missing socio-demographic features in Table 1 (Nationality and Children). Since the participants of this survey were all students with similar age (97% of the participants had an age between 22 and 30 years), we think that age should be properly expressed as mean and SD rather than categorizing it as age classes.

3. **In the results section, the titles of all the five tables presented in the paper ought to have been typed at the top of the tables contrary to the bottom of the tables as presently presented by the authors. This needs to be corrected.**

We appreciated this comment and we corrected the titles of the tables as suggested.

**Rev 4:**

1. **Research regarding gender-based differential patterns of care by health providers arise predominantly from the developed countries. In my opinion, similar studies from developing countries are also important to be compared. Could you elaborate more on this issue? For example a study below presents the role that gender has on health. Fikree, F. F., & Pasha, O. (2004). Role of gender in health disparity: the South Asian context. BMJ (Clinical research ed.), 328(7443), 823 826.** [**https://doi.org/10.1136/bmj.328.7443.823**](https://doi.org/10.1136/bmj.328.7443.823)

Thanks for your comment, we added this part in the discussion section: “It is important to consider that the European context is in any case much better in terms of gender inequalities in health than in developing countries (24).”

We think that it is difficult to compare our results with studies from developing countries. There are no data in the literature on gender awareness in medical students in developing countries.

2. **Page 5, the questionnaire section: After a review of the literature, a 29-items questionnaire was developed. Please be more detail on how to develop the questionnaire through the literature review.**

Thank you for this comment. As also requested by Reviewer n°2, we added the following paragraph describing the variables analyzed and the construction of the questionnaire.

Table 1 displays the variables assessed to describe the sample, stratified by gender. In particular, we explored the following characteristics: age, year of course, nationality, marital status, having children, self-perceived health status, having one or more chronic diseases, familiarity for chronic diseases. Self-perceived health status was assessed with a Likert scale, ranging from 1 (very bad) to 5 (very good), and it was subsequently dichotomized in “not good” (score 1 and 2) and “good/very good” (score 3, 4 and 5). Gender medicine knowledge was assessed with questions regarding the correct definitions of sex, gender and gender medicine, gender-related epidemiology of frequent diseases, and true/false questions regarding specific gender medicine issues.

Personal experience during academic training were assessed asking the students whether they have dealt with gender medicine issues during lessons and during traineeships in the wards”.

As specified in the text, the only part of the questionnaire derived from the literature was the N-GAMS scale, for which we provided the appropriate citation.

3. **This study used survey instrument- a questionnaire containing the N-GAMS scale to measure gender awareness. Is it in italian-language questionnaire? If yes, how the translation process and validation?**

We appreciate this comment. We add this sentence: “The questionnaire was in Italian-language. A bilingual researcher translate the tool from English to Italian. The translated N-GAMS scale was then tested for understandability in a pilot study recruiting 20 medical students.”

4. **Kindly indicate the reasons why students of the fifth and sixth years and outside prescribed time were chosen for this study.**

We appreciate this comment. We completed the text in the Material and Methods section as follows: “The questionnaire was submitted to students of the last years of the degree course because they already had practical training experiences with patients and had received an important part of theoretical training”.

**Rev 5:**

1. **In many diseases, such as coronary artery disease, Parkinson disease, irritable bowel
   syndrome, neck pain, knee joint arthrosis and tuberculosis, men are treated more extensively than women (8). Research found that physicians are more likely to consider symptoms reported by men as organic (8). Gender awareness in doctors aims to equity and equality in health. Did you mean to say these diseases are treated more extensively by men than women? Kindly explain and paraphrase your sentence properly.**

Thank you for your consideration. We added information about this as follows:

In many diseases, such as coronary artery disease, Parkinson disease, irritable bowel syndrome, neck pain, knee joint arthrosis and tuberculosis, men are treated more extensively than women (8). Research found that physicians are more likely to consider symptoms reported by men as organic (8). These differences, if not properly considered, can lead to health inequality. In particular, some pathologies can be undertreated in women compared to men or vice versa. For this reason, it is important that doctors are aware of gender differences during their clinical practice.
