# Supplementary material for: Gender sensitivity and stereotypes in medical university students: An Italian cross-sectional study
Source: PLoS One. 2022 Jan 6;17(1):e0262324. doi: 10.1371/journal.pone.0262324 (PMC8735594; doi:10.1371/journal.pone.0262324)
Supplement: Attachment — Submitted filename: DR AJOKE REVIEW PLOS.docx [file pone.0262324.s004.docx]

PLOS ONE: Are 2020’s medical students still suffering from gender stereotypes? An Italian crossectional

study

--Manuscript Draft--

| **Pages** | **Queries** | **My suggestions to the author** |
| --- | --- | --- |
| 1 | Title issue | Very good article but my concern is that a research question cannot be a title.  Kindly consider and change the title into a statement and not a question, the question can be included as part of your introduction, along with your aim of study |
| 4 | Data absent | Kindly include the data to reveal instances about gender differences influencing health.  Please state clearly the impact (state wise, nationally and globally) and effect on people’s health, physically, socially, mentally e.t.c  Which is one of the reasons why you must have chosen the topic. |
|  | Study Rationale | Invariably including a strong rationale for this study would have brought out the best of this article |
|  |  |  |
