# Supplementary material for: Gender sensitivity and stereotypes in medical university students: An Italian cross-sectional study
Source: PLoS One. 2022 Jan 6;17(1):e0262324. doi: 10.1371/journal.pone.0262324 (PMC8735594; doi:10.1371/journal.pone.0262324)
Supplement: Attachment — Submitted filename: Response to Reviewers .docx [file pone.0262324.s006.docx]

Dear Editor,

We are submitting the revised version of our manuscript **“Are 2020’s medical students still suffering from gender stereotypes? An Italian cross-sectional study”**, according to the comments of the reviewers.

**Rev 1:**

**1 Title issue Very good article but my concern is that a research question cannot be a title.**

**Kindly consider and change the title into a statement and not a question, the question can be included as part of your introduction, along with your aim of study**

**4 Data absent Kindly include the data to reveal instances about gender differences influencing health.**

**Please state clearly the impact (state wise, nationally and globally) and effect on people’s health, physically, socially, mentally e.t.c**

**Which is one of the reasons why you must have chosen the topic.**

**Study Rationale Invariably including a strong rationale for this study would have brought out the best of this article**

We would like to thank you for your useful considerations.

We modified title: “Gender sensitivity and stereotypes in medical university students: An Italian cross-sectional study”.

We have also modified the introduction as follows:

“A Danish study with a sample of 6.9 million patients, reports that women receive a diagnosis of disease 4 years later than men(8). Another study reports that the in-hospital mortality of an acute myocardial infarction (AMI) is higher in women than in men up to 70 years of age and survival after 6 months of AMI is lower in women(9).”

“The focus on a medicine that takes gender differences into account begins in the 80s with the signing by the UN (United Nation) of a convention aimed at eliminating all forms of discrimination against women(22). In Europe, a growing interest in gender medicine led to the inclusion of this issue in the new Horizon 2020 research funding program(22). In Italy, a *Plan for the application and diffusion of Gender Specific Medicine* (2019) aims to spread the principles of gender specific medicine that are not yet fully and adequately implemented in medical academic training (22). For this reason, the aim of this study is to evaluate gender awareness through N-GAMS scale and its predictors in Italian male and female medical students, since no data exist in literature about gender awareness in our national context.”

Finally, we want to thank the other reviewers for their helpful consideration.
